# Supplementary material for: Relative fertility of HIV-positive women in the ART era: updated estimates from national household survey data
Source: medRxiv. 2026 Aug 12:2026.08.11.26360197. Preprint. [Version 1] doi: 10.64898/2026.08.11.26360197 (PMC13640869; doi:10.64898/2026.08.11.26360197)
Supplement: Supplementary file 1 [file NIHPP2026.08.11.26360197v1-supplement-1.pdf]

medRxiv preprint doi: https://doi.org/10.64898/2026.08.11.26360197; this version posted August 12, 2026. The copyright holder for this preprint (which was not certified by peer review) is the author/funder, who has granted medRxiv a license to display the preprint in perpetuity.
It is made available under a CC-BY 4.0 International license .

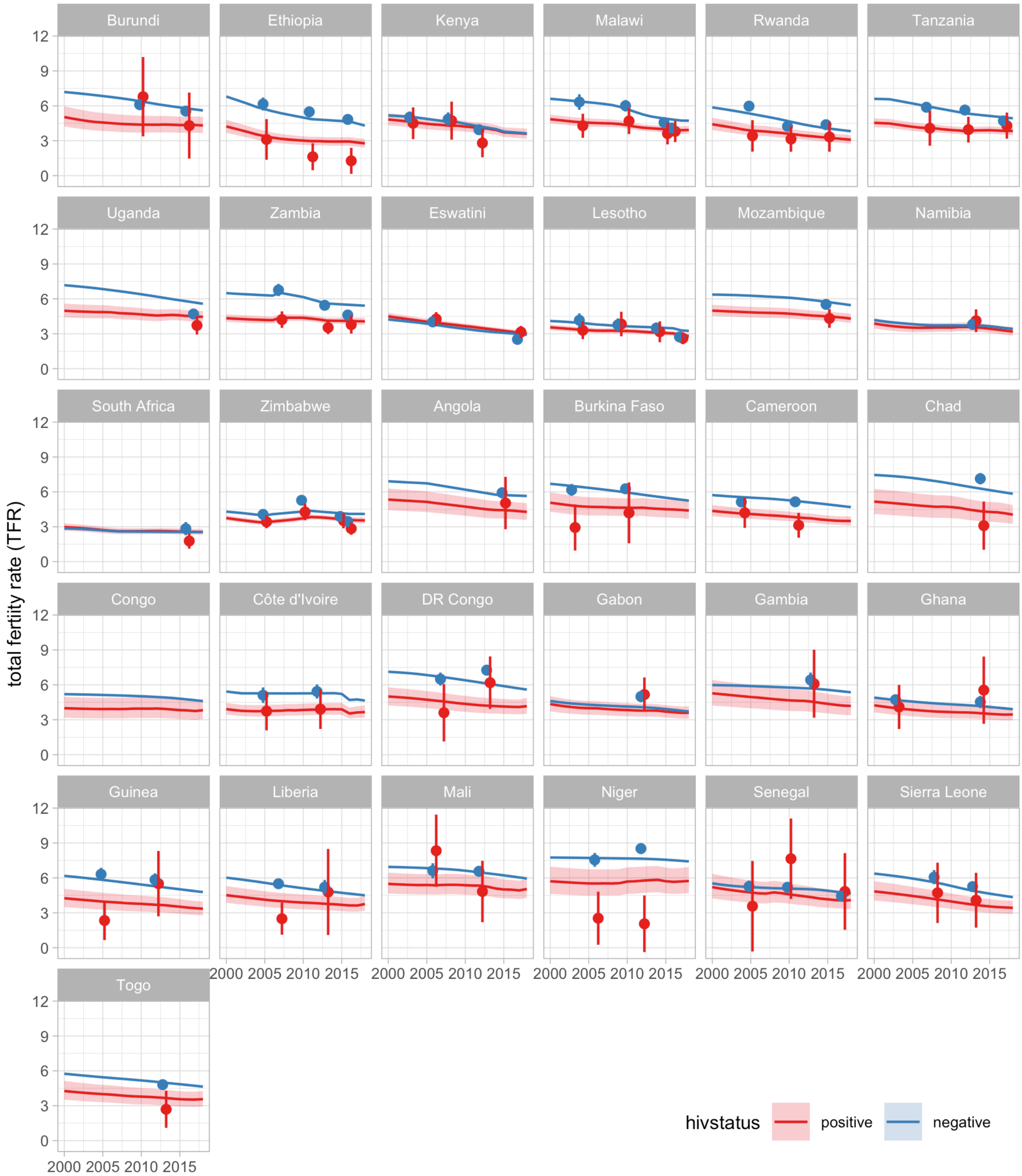


**Figure S1:** Predicted and observed total fertility rate (TFR) by HIV status. Points and vertical lines reflect direct survey estimates and 95% confidence intervals for TFR by HIV status in the 12 months preceding the survey from 72 household surveys analysed (Table 1). Estimates for total TFR (both HIV-postive and HIV-negative) and age-specific HIV prevalence are taken from Spectrum 2018 point estimates; and estimates for relative fertility of HIV positive women are from the present analysis.

medRxiv preprint doi: https://doi.org/10.64898/2026.08.11.26360197; this version posted August 12, 2026. The copyright holder for this preprint (which was not certified by peer review) is the author/funder, who has granted medRxiv a license to display the preprint in perpetuity.
It is made available under a CC-BY 4.0 International license .

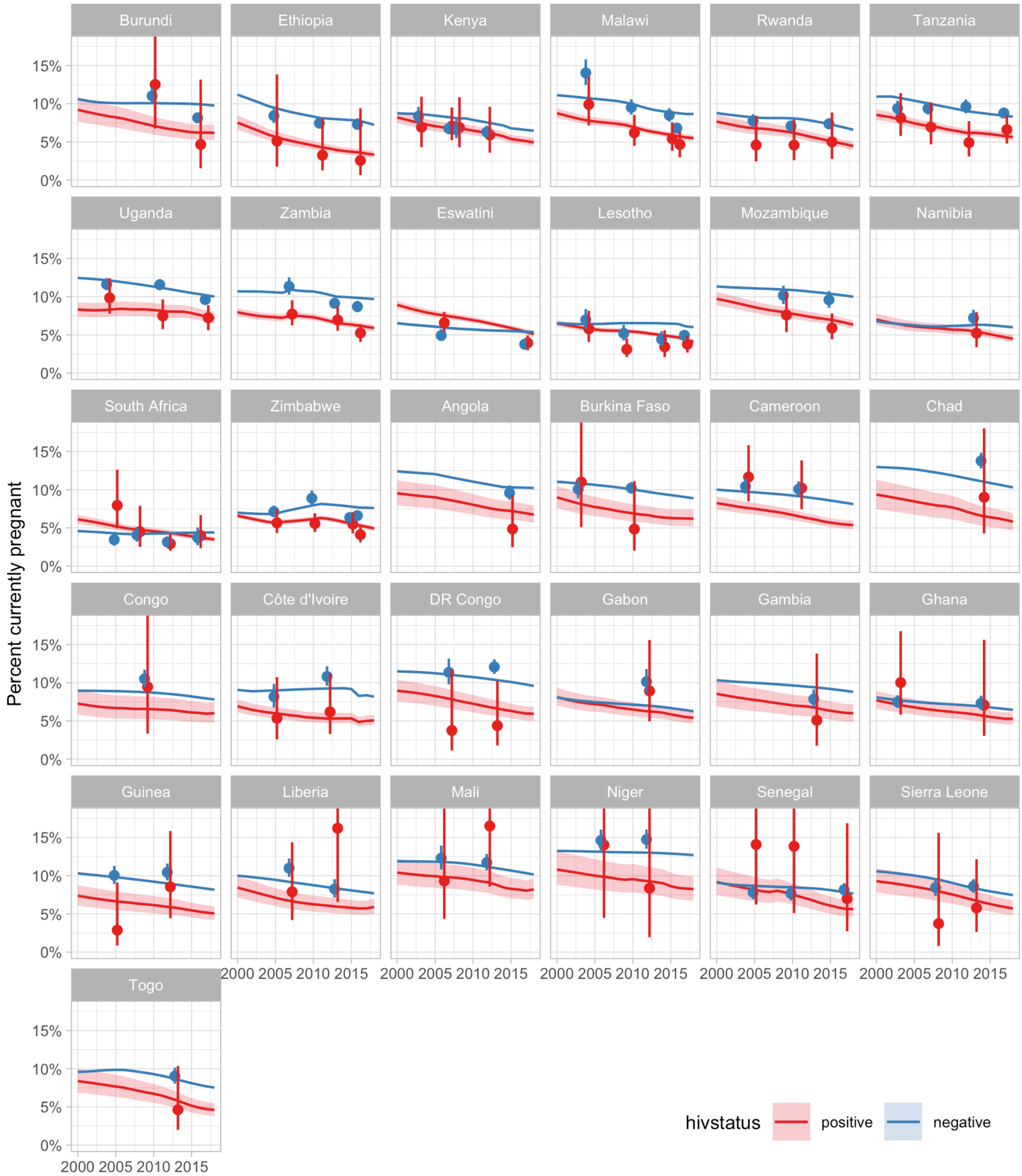


**Figure S2:** Predicted and observed percent currently pregnant by HIV status. Points and vertical lines reflect direct survey estimates and 95% confidence intervals for the percentage of women self-reporting being currently pregnant at the time of survey by HIV status from 72 household surveys analysed (Table 1). Estimates for total ASFR (both HIV-postive and HIV-negative) and age-specific HIV prevalence are taken from Spectrum 2018 estimates. Estimates for relative fertility of HIV positive women are from the present analysis.

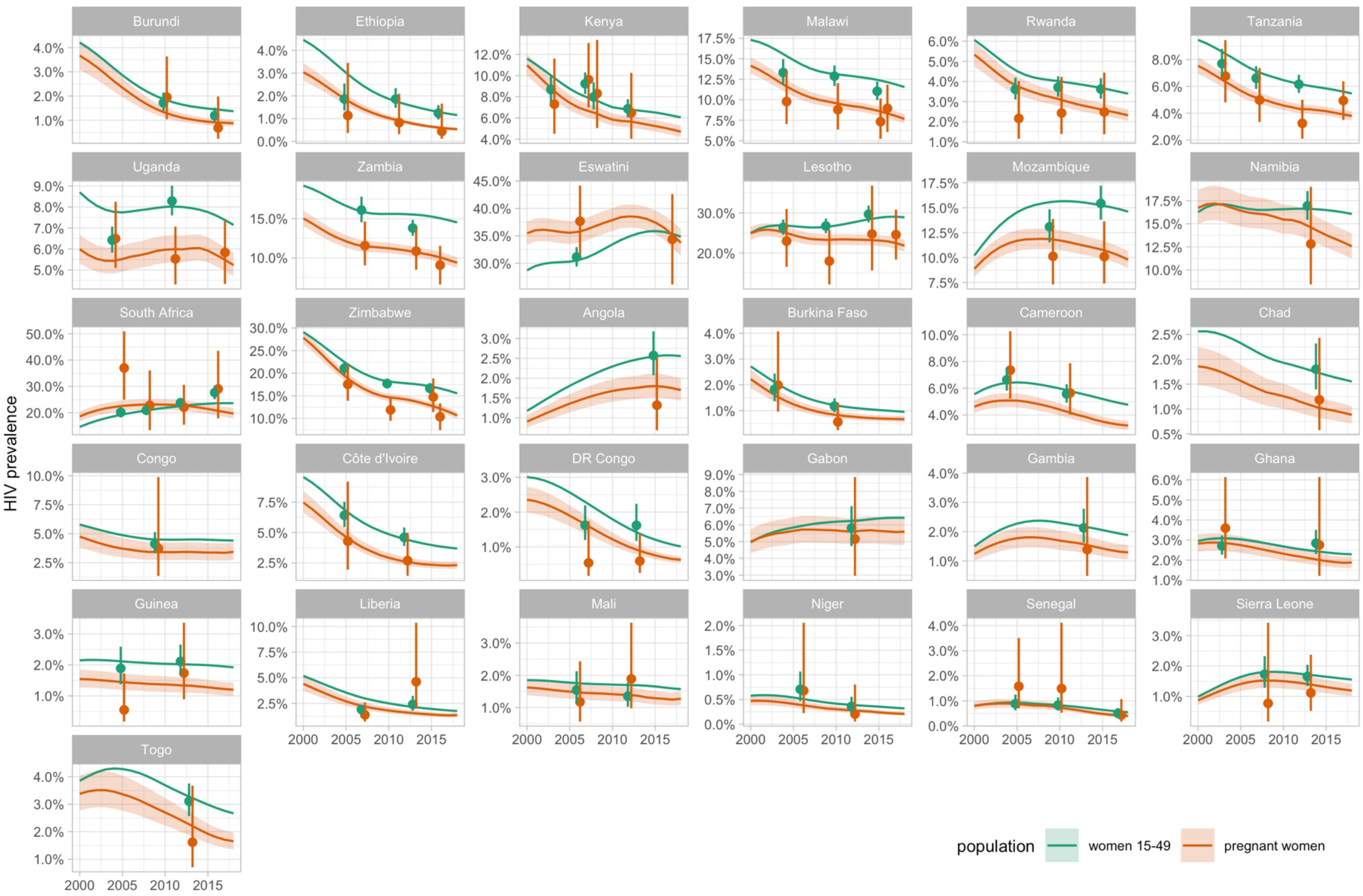


**Figure S3:** Predicted and observed HIV prevalence among all women aged 15-49 years and currently pregnant women. Points and vertical lines reflect direct survey estimates and 95% confidence intervals for HIV prevalence among all women aged 15-49 and women self-reporting being currently pregnant. Estimates for prevalence age 15-49 are taken from Spectrum 2018 estimates; estimates for prevalence among pregnant women are calculated from relative fertility rates estimated in the present analysis.
